# Supplementary material for: Facing the COVID-19 pandemic inside maternities in Brazil: A mixed-method study within the REBRACO initiative
Source: PLoS One. 2021 Jul 23;16(7):e0254977. doi: 10.1371/journal.pone.0254977 (PMC8301675; doi:10.1371/journal.pone.0254977)
Supplement: S1 Data — List of variables which were collected through online forms. (DOCX) [file pone.0254977.s002.docx]

**Supporting information-S1**

**S1: Variables requested for the characterization of the included REBRACO centers.** List of variables which were collected through online forms

| **Infrastructure/Equipment** |
| --- |
| Number of beds designated for labor ward (per month, from March to August) |
| Number of beds designated for obstetric medical ward (per month, from March to August) |
| Number of beds designated for intensive care (per month, from March to August) |
| Availability of the tests to confirm the infection by SARS-CoV 2 and number of tested women (per month, from March to August) |
| Implementation of modifications in the maternity´s infra-structure (resizing wards, relocation of beds, acquiring new equipments, such as ventilators)  Availability of PPE (Personal Protective Equipment) |
|  |
| **Maternal and perinatal health indicators** |
| Total number of attendances at the obstetric emergency care unit (per month, from March to August) |
| Number of attendances at the obstetric emergency care unit (per month, from March to August) for pregnant and postpartum women with suspected COVID-19 |
| Number of new confirmed cases of COVID-19 in pregnant and postpartum women (per month, from March to August) |
| Criteria for suspected cases of COVID-19 |
| Number of hospitalizations of pregnant and postpartum women in the unit (per month, from March to August) |
| Number of hospitalizations due to COVID-19 in pregnant and postpartum women in the unit (per month, from March to August) (suspected or confirmed) |
| Number of new cases of severe acute respiratory syndrome (SARS) in pregnant/postpartum women, in the unit (per month, from March to August) |
| Number of new cases of severe acute respiratory syndrome (SARS) caused by COVID-19 in pregnant/postpartum women, in the unit (per month, from March to August) |
| Number of maternal deaths due to all causes in the unit (per month, from March to August) |
| Number of maternal deaths due to COVID-19 unit (per month, from March to August) |
| Number of live births in you unit (per month, from March to August) |
| Cesarean section rate (%; elective or intrapartum cesarean) in the unit (per month, from March to August) |
| Number of live births in the municipality unit (per month, from March to August) |
| Number of stillbirths in the unit (per month, from March to August) |
| Number of stillbirths in the municipality unit (per month, from March to August) |
|  |
| **Other characteristics of service provision** |
| Referral center for cases suspected/confirmed for COVID-19 (y/n) |
| Rules on the presence of companions during childbirth (always, often, sometimes, rarely or never; per month, from March to August) |
| How long on average was the turnover to obtain test results for COVID-19? (in days; per month, from March to August) |
| Gynecological and obstetrical services according to the usual standard in the unit (suspended, reduction, no change, not applicable) |
|  |
| **Staff/Human Resources** |
| Presence of Ob&Gyn medical residents (y/n) |
| Number of professionals that received sick leave due to COVID-19 in the unit (per month, from March to August) |
| Members of the COVID-19 Emergency Action Committee (EAC) - (according to type of health professionals) and date of EAC implementation (from January to August) |
| Modification of work schedule of health professionals (Hiring new professionals, increase in weekly workload, suspension of annual leaves) |
| Content of the training program for COVID-19 |
