# Supplementary material for: Facing the COVID-19 pandemic inside maternities in Brazil: A mixed-method study within the REBRACO initiative
Source: PLoS One. 2021 Jul 23;16(7):e0254977. doi: 10.1371/journal.pone.0254977 (PMC8301675; doi:10.1371/journal.pone.0254977)
Supplement: S2 Data — Information about the organization, health care and consolidated results of the REBRACO participating centers (from March to August). (DOCX) [file pone.0254977.s003.docx]

**Supporting information-S2**

**S1: Data collection form.** Information about the organization, health care and consolidated results of the REBRACO participating centers (from March to August).

**Data collection form – English Version (translated)**

**Management Team – Gathering information about the organization, health care and consolidated results of the REBRACO participating centers.**

*Information from March to August.*

1. Research center:

Caism/Unicamp, Campinas

Hospital Estadual de Sumaré - HES, Sumaré

Hospital Universitário/FMJ - Jundiaí

HC/UFPE, Recife

MEAC/UFC, Fortaleza

UNIFESP/EPM, São Paulo

FMRP-USP, Ribeirão Preto

HC/UFRGS, Porto Alegre

Instituto Fernandes Figueira - IFF/Fiocruz, Rio de Janeiro

Hospital Moinhos de Vento, Porto Alegre

Hospital UNIMED

Santa Casa de São Carlos/UFSCAR, São Carlos

Maternidade Climério de Oliveira-UFBA, Salvador

Hospital Regional Jorge Rossmann, Itanhaém

Universidade Federal de Minas Gerais - HC/UFMG, Belo Horizonte

Faculdade de Medicina da Universidade Estadual de São Paulo - FMB/UNESP, Botucatu

1. Does your unit have residents in obstetrics and gynecology?

Yes

No

1. Total number of attendances at the obstetric emergency care unit: (pregnant and postpartum women)

- March:

- April:

- May:

- June:

- July:

- August:

1. Number of attendances at the obstetric emergency care unit for pregnant and postpartum women with suspected COVID-19:

- March:

- April:

- May:

- June:

- July:

- August:

1. Number of new confirmed cases of COVID-19 in pregnant and postpartum women:

- March:

- April:

- May:

- June:

- July:

- August:

1. Criteria for suspected cases of COVID-19 include:

Fever

Cough

Fatigue

Sore Throat

Conjunctivitis

Nasal congestion

Coryza

Headache

Loss of taste

Loss of smell

Skin rash

Diarrhea

Abdominal pain

Dyspnea

Chest pain

1. Number of hospitalizations of pregnant and postpartum women in your unit:

- March:

- April:

- May:

- June:

- July:

- August:

1. Number of hospitalizations due to COVID-19 in pregnant and postpartum women in your unit (suspected or confirmed):

- March:

- April:

- May:

- June:

- July:

- August:

1. Number of new cases of severe acute respiratory syndrome (SARS) in pregnant women, in your unit:

- March:

- April:

- May:

- June:

- July:

- August:

1. Number of new cases of severe acute respiratory syndrome (SARS) caused by COVID-19 in pregnant women, in your unit:

- March:

- April:

- May:

- June:

- July:

- August:

1. Number of maternal deaths due to all causes:

- March:

- April:

- May:

- June:

- July:

- August:

1. Number of maternal deaths due to COVID-19:

- March:

- April:

- May:

- June:

- July:

- August:

1. Number of professional that received sick leave due to COVID-19 in the unit:

- March:

- April:

- May:

- June:

- July:

- August:

1. Number of beds designated for labor ward:

- March:

- April:

- May:

- June:

- July:

- August:

1. Number of beds designated for rooming:

- March:

- April:

- May:

- June:

- July:

- August:

1. Number of beds designated for intensive care:

- March:

- April:

- May:

- June:

- July:

- August:

1. Number of live births in you unit:

- March:

- April:

- May:

- June:

- July:

- August:

1. Cesarean rate (elective or intrapartum cesarean) in %:

- March:

- April:

- May:

- June:

- July:

- August:

1. Number of live births in your municipality:

- March:

- April:

- May:

- June:

- July:

- August:

1. Number of fetal deaths in your unit:

- March:

- April:

- May:

- June:

- July:

- August:

1. Number of fetal deaths in your municipality:

- March:

- April:

- May:

- June:

- July:

- August:

1. As for gynecology assistance clinics: (*make only one check per line per month)*

|  | **Reduction in the supply of care** | **Stoppaage of service provision** | **There was no change** | **Not applicable (there is no such service)** |
| --- | --- | --- | --- | --- |
| **General outpatient clinic** | \| **March** \| **June** \| \| --- \| --- \| \| **April** \| **July** \| \| **May** \| **August** \| | \| **March** \| **June** \| \| --- \| --- \| \| **April** \| **July** \| \| **May** \| **August** \| | \| **March** \| **June** \| \| --- \| --- \| \| **April** \| **July** \| \| **May** \| **August** \| | \| **March** \| **June** \| \| --- \| --- \| \| **April** \| **July** \| \| **May** \| **August** \| |
| **Videolaparoscopy** | \| **March** \| **June** \| \| --- \| --- \| \| **April** \| **July** \| \| **May** \| **August** \| | \| **March** \| **June** \| \| --- \| --- \| \| **April** \| **July** \| \| **May** \| **August** \| | \| **March** \| **June** \| \| --- \| --- \| \| **April** \| **July** \| \| **May** \| **August** \| | \| **March** \| **June** \| \| --- \| --- \| \| **April** \| **July** \| \| **May** \| **August** \| |
| **Infectious diseases of the lower genital tract** | \| **March** \| **June** \| \| --- \| --- \| \| **April** \| **July** \| \| **May** \| **August** \| | \| **March** \| **June** \| \| --- \| --- \| \| **April** \| **July** \| \| **May** \| **August** \| | \| **March** \| **June** \| \| --- \| --- \| \| **April** \| **July** \| \| **May** \| **August** \| | \| **March** \| **June** \| \| --- \| --- \| \| **April** \| **July** \| \| **May** \| **August** \| |
| **Urogynecology** | \| **March** \| **June** \| \| --- \| --- \| \| **April** \| **July** \| \| **May** \| **August** \| | \| **March** \| **June** \| \| --- \| --- \| \| **April** \| **July** \| \| **May** \| **August** \| | \| **March** \| **June** \| \| --- \| --- \| \| **April** \| **July** \| \| **May** \| **August** \| | \| **March** \| **June** \| \| --- \| --- \| \| **April** \| **July** \| \| **May** \| **August** \| |
| **Contraception** | \| **March** \| **June** \| \| --- \| --- \| \| **April** \| **July** \| \| **May** \| **August** \| | \| **March** \| **June** \| \| --- \| --- \| \| **April** \| **July** \| \| **May** \| **August** \| | \| **March** \| **June** \| \| --- \| --- \| \| **April** \| **July** \| \| **May** \| **August** \| | \| **March** \| **June** \| \| --- \| --- \| \| **April** \| **July** \| \| **May** \| **August** \| |

1. As for obstetrics assistance clinics: (*make only one check per line per month)*

|  | **Reduction of provision of care** | **Stoppaage of service provision** | **No change on the provision of care** | **Not applicable (there is no such service)** |
| --- | --- | --- | --- | --- |
| **Low-risk antenatal care** | \| **March** \| **June** \| \| --- \| --- \| \| **April** \| **July** \| \| **May** \| **August** \| | \| **March** \| **June** \| \| --- \| --- \| \| **April** \| **July** \| \| **May** \| **August** \| | \| **March** \| **June** \| \| --- \| --- \| \| **April** \| **July** \| \| **May** \| **August** \| | \| **March** \| **June** \| \| --- \| --- \| \| **April** \| **July** \| \| **May** \| **August** \| |
| **High-risk antenatal care** | \| **March** \| **June** \| \| --- \| --- \| \| **April** \| **July** \| \| **May** \| **August** \| | \| **March** \| **June** \| \| --- \| --- \| \| **April** \| **July** \| \| **May** \| **August** \| | \| **March** \| **June** \| \| --- \| --- \| \| **April** \| **July** \| \| **May** \| **August** \| | \| **March** \| **June** \| \| --- \| --- \| \| **April** \| **July** \| \| **May** \| **August** \| |
| **Postpartum outpatient clinic** | \| **March** \| **June** \| \| --- \| --- \| \| **April** \| **July** \| \| **May** \| **August** \| | \| **March** \| **June** \| \| --- \| --- \| \| **April** \| **July** \| \| **May** \| **August** \| | \| **March** \| **June** \| \| --- \| --- \| \| **April** \| **July** \| \| **May** \| **August** \| | \| **March** \| **June** \| \| --- \| --- \| \| **April** \| **July** \| \| **May** \| **August** \| |
| **Obstetrics imaging service (Ultrasound, etc)** | \| **March** \| **June** \| \| --- \| --- \| \| **April** \| **July** \| \| **May** \| **August** \| | \| **March** \| **June** \| \| --- \| --- \| \| **April** \| **July** \| \| **May** \| **August** \| | \| **March** \| **June** \| \| --- \| --- \| \| **April** \| **July** \| \| **May** \| **August** \| | \| **March** \| **June** \| \| --- \| --- \| \| **April** \| **July** \| \| **May** \| **August** \| |
| **Clinical analysis laboratory** | \| **March** \| **June** \| \| --- \| --- \| \| **April** \| **July** \| \| **May** \| **August** \| | \| **March** \| **June** \| \| --- \| --- \| \| **April** \| **July** \| \| **May** \| **August** \| | \| **March** \| **June** \| \| --- \| --- \| \| **April** \| **July** \| \| **May** \| **August** \| | \| **March** \| **June** \| \| --- \| --- \| \| **April** \| **July** \| \| **May** \| **August** \| |

1. How do you consider suitability on the following topics? In your center, and in each specific month.

|  | **Enough for all units** | **Enough for most units** | **Insufficient for most** | **Insufficient for all units** |
| --- | --- | --- | --- | --- |
| **Provision of personal protective equipment** | \| **March** \| **June** \| \| --- \| --- \| \| **April** \| **July** \| \| **May** \| **August** \| | \| **March** \| **June** \| \| --- \| --- \| \| **April** \| **July** \| \| **May** \| **August** \| | \| **March** \| **June** \| \| --- \| --- \| \| **April** \| **July** \| \| **May** \| **August** \| | \| **March** \| **June** \| \| --- \| --- \| \| **April** \| **July** \| \| **May** \| **August** \| |
| **Team training** | \| **March** \| **June** \| \| --- \| --- \| \| **April** \| **July** \| \| **May** \| **August** \| | \| **March** \| **June** \| \| --- \| --- \| \| **April** \| **July** \| \| **May** \| **August** \| | \| **March** \| **June** \| \| --- \| --- \| \| **April** \| **July** \| \| **May** \| **August** \| | \| **March** \| **June** \| \| --- \| --- \| \| **April** \| **July** \| \| **May** \| **August** \| |
| **Supporting material to guide flows, decisions and procedures.** | \| **March** \| **June** \| \| --- \| --- \| \| **April** \| **July** \| \| **May** \| **August** \| | \| **March** \| **June** \| \| --- \| --- \| \| **April** \| **July** \| \| **May** \| **August** \| | \| **March** \| **June** \| \| --- \| --- \| \| **April** \| **July** \| \| **May** \| **August** \| | \| **March** \| **June** \| \| --- \| --- \| \| **April** \| **July** \| \| **May** \| **August** \| |

1. In general, the entry of companions during childbirth car and care for the newborn was (make only one per line per month):

|  | **Always** | **Often** | **Sometimes** | **Rarely** | **Never** |
| --- | --- | --- | --- | --- | --- |
| **During labor** | \| **March** \| **June** \| \| --- \| --- \| \| **April** \| **July** \| \| **May** \| **August** \| | \| **March** \| **June** \| \| --- \| --- \| \| **April** \| **July** \| \| **May** \| **August** \| | \| **March** \| **June** \| \| --- \| --- \| \| **April** \| **July** \| \| **May** \| **August** \| | \| **March** \| **June** \| \| --- \| --- \| \| **April** \| **July** \| \| **May** \| **August** \| | \| **March** \| **June** \| \| --- \| --- \| \| **April** \| **July** \| \| **May** \| **August** \| |
| **At delivery** | \| **March** \| **June** \| \| --- \| --- \| \| **April** \| **July** \| \| **May** \| **August** \| | \| **March** \| **June** \| \| --- \| --- \| \| **April** \| **July** \| \| **May** \| **August** \| | \| **March** \| **June** \| \| --- \| --- \| \| **April** \| **July** \| \| **May** \| **August** \| | \| **March** \| **June** \| \| --- \| --- \| \| **April** \| **July** \| \| **May** \| **August** \| | \| **March** \| **June** \| \| --- \| --- \| \| **April** \| **July** \| \| **May** \| **August** \| |
| **At the rooming** | \| **March** \| **June** \| \| --- \| --- \| \| **April** \| **July** \| \| **May** \| **August** \| | \| **March** \| **June** \| \| --- \| --- \| \| **April** \| **July** \| \| **May** \| **August** \| | \| **March** \| **June** \| \| --- \| --- \| \| **April** \| **July** \| \| **May** \| **August** \| | \| **March** \| **June** \| \| --- \| --- \| \| **April** \| **July** \| \| **May** \| **August** \| | \| **March** \| **June** \| \| --- \| --- \| \| **April** \| **July** \| \| **May** \| **August** \| |
| **At the neonatal ICU** | \| **March** \| **June** \| \| --- \| --- \| \| **April** \| **July** \| \| **May** \| **August** \| | \| **March** \| **June** \| \| --- \| --- \| \| **April** \| **July** \| \| **May** \| **August** \| | \| **March** \| **June** \| \| --- \| --- \| \| **April** \| **July** \| \| **May** \| **August** \| | \| **March** \| **June** \| \| --- \| --- \| \| **April** \| **July** \| \| **May** \| **August** \| | \| **March** \| **June** \| \| --- \| --- \| \| **April** \| **July** \| \| **May** \| **August** \| |

1. The tests to confirm infection with the SARS-CoV 2 virus were: (check all that apply each month)

***March***

Performed in the hospital laboratory

Performed in a partner laboratory (external service)

Instituto Adolfo Lutz (Brazilian National laboratory)

State laboratory

Municipal laboratory

***April***

Performed in the hospital laboratory

Performed in a partner laboratory (external service)

Instituto Adolfo Lutz (Brazilian National laboratory)

State laboratory

Municipal laboratory

***May***

Performed in the hospital laboratory

Performed in a partner laboratory (external service)

Instituto Adolfo Lutz (Brazilian National laboratory)

State laboratory

Municipal laboratory

***June***

Performed in the hospital laboratory

Performed in a partner laboratory (external service)

Instituto Adolfo Lutz (Brazilian National laboratory)

State laboratory

Municipal laboratory

***July***

Performed in the hospital laboratory

Performed in a partner laboratory (external service)

Instituto Adolfo Lutz (Brazilian National laboratory)

State laboratory

Municipal laboratory

***August***

Performed in the hospital laboratory

Performed in a partner laboratory (external service)

Instituto Adolfo Lutz (Brazilian National laboratory)

State laboratory

Municipal laboratory

1. How long on average used to take the test results? (in days)

- March:

- April:

- May:

- June:

- July:

- August:

1. Whats strategies were taken in place to monitor cases of suspected and / or confirmed cases of COVID-19: (check all that apply each month)

***March***

Telephone contact (calls)

Message contact

Video call

Follow up in the outpatient clinic in the same service

There was no strategy

Other – Specify:

***April***

Telephone contact (calls)

Message contact

Video call

Follow up in the outpatient clinic in the same service

There was no strategy

Other – Specify:

***May***

Telephone contact (calls)

Message contact

Video call

Follow up in the outpatient clinic in the same service

There was no strategy

Other – Specify:

***June***

Telephone contact (calls)

Message contact

Video call

Follow up in the outpatient clinic in the same service

There was no strategy

Other – Specify:

***July***

Telephone contact (calls)

Message contact

Video call

Follow up in the outpatient clinic in the same service

There was no strategy

Other – Specify:

***August***

Telephone contact (calls)

Message contact

Video call

Follow up in the outpatient clinic in the same service

There was no strategy

Other – Specify:

**Data collection form – Portuguese Version (Original)**

**Comitê Gestor - Coleta de Informações sobre a Organização, Assistência e Resultados Consolidados dos Centros Participantes ReBraCO.**

*Informações referentes aos meses de março, abril, maio, junho, julho e agosto.*

1. Centro Colaborador:

Caism/Unicamp, Campinas

Hospital Estadual de Sumaré - HES, Sumaré

Hospital Universitário/FMJ - Jundiaí

HC/UFPE, Recife

MEAC/UFC, Fortaleza

UNIFESP/EPM, São Paulo

FMRP-USP, Ribeirão Preto

HC/UFRGS, Porto Alegre

Instituto Fernandes Figueira - IFF/Fiocruz, Rio de Janeiro

Hospital Moinhos de Vento, Porto Alegre

Hospital UNIMED, Belo Horizonte

Santa Casa de São Carlos/UFSCAR, São Carlos

Maternidade Climério de Oliveira-UFBA, Salvador

Hospital Regional Jorge Rossmann, Itanhaém

Universidade Federal de Minas Gerais - HC/UFMG, Belo Horizonte

Faculdade de Medicina da Universidade Estadual de São Paulo - FMB/UNESP, Botucatu

1. Sua unidade possui residentes em Ginecologia e Obstetrícia?

Sim

Não

1. Número total de atendimentos no serviço de pronto-atendimento em obstetrícia: (Gestantes e Puérperas)

- Março:

- Abril:

- Maio:

- Junho:

- Julho:

- Agosto:

1. Número de atendimentos no serviço de pronto-atendimento de gestantes ou puérperas com suspeita de COVID-19:

- Março:

- Abril:

- Maio:

- Junho:

- Julho:

- Agosto:

1. Número de novos casos de COVID-19 confirmados em gestante ou puérperas:

- Março:

- Abril:

- Maio:

- Junho:

- Julho:

- Agosto:

1. Critérios para suspeição COVID-19 incluem:

Febre

Tosse

Cansaço

Dor de garganta

Conjuntivite

Congestão nasal

Coriza

Cefaléia

Perda de paladar (qualquer grau

Perda de olfato (qualquer grau)

Erupção cutânea

Diarréia

Dor Abdominal

Falta de ar ou dificuldade para respirar

Dor no peito

1. Número de internações hospitalares de gestantes ou puérperas na unidade:

- Março:

- Abril:

- Maio:

- Junho:

- Julho:

- Agosto:

1. Número de internações hospitalares por COVID-19 em gestantes ou puérperas na unidade (suspeita ou confirmado):

- Março:

- Abril:

- Maio:

- Junho:

- Julho:

- Agosto:

1. Número de novos casos de síndrome respiratória aguda grave (SRAG) em gestantes na unidade:

- Março:

- Abril:

- Maio:

- Junho:

- Julho:

- Agosto:

1. Número de novos casos de síndrome respiratória aguda grave (SRAG) por COVID-19 em gestantes na unidade:

- Março:

- Abril:

- Maio:

- Junho:

- Julho:

- Agosto:

1. Número de óbitos maternos por qualquer causa:

- Março:

- Abril:

- Maio:

- Junho:

- Julho:

- Agosto:

1. Número de óbitos maternos por COVID-19:

- Março:

- Abril:

- Maio:

- Junho:

- Julho:

- Agosto:

1. Número de afastamentos de profissionais de saúde devido COVID-19 na unidade:

- Março:

- Abril:

- Maio:

- Junho:

- Julho:

- Agosto:

1. Número de leitos designados para pré-parto:

- Março:

- Abril:

- Maio:

- Junho:

- Julho:

- Agosto:

1. Número de leitos designados para alojamento conjunto:

- Março:

- Abril:

- Maio:

- Junho:

- Julho:

- Agosto:

1. Número de leitos designados para cuidados intensivos:

- Março:

- Abril:

- Maio:

- Junho:

- Julho:

- Agosto:

1. Número de nascidos vivos na sua unidade:

- Março:

- Abril:

- Maio:

- Junho:

- Julho:

- Agosto:

1. Taxa de cesárea (cesárea eletiva ou intraparto) em %:

- Março:

- Abril:

- Maio:

- Junho:

- Julho:

- Agosto:

1. Número de nascidos vivos no seu município:

- Março:

- Abril:

- Maio:

- Junho:

- Julho:

- Agosto:

1. Número de óbitos fetais atendidos na sua unidade:

- Março:

- Abril:

- Maio:

- Junho:

- Julho:

- Agosto:

1. Número de óbitos fetais no seu município:

- Março:

- Abril:

- Maio:

- Junho:

- Julho:

- Agosto:

1. Quanto aos ambulatórios de assistência em ginecologia: (*marcar apenas um check por linha por mês)*

|  | **Redução da oferta de atendimentos** | **Paralisação na oferta de atendimentos** | **Não houve Modificação na oferta de atendimentos** | **Não se aplica (não há esse serviço no centro** |
| --- | --- | --- | --- | --- |
| **Ambulatório Geral** | \| **Março** \| **Junho** \| \| --- \| --- \| \| **Abril** \| **Julho** \| \| **Maio** \| **Agosto** \| | \| **Março** \| **Junho** \| \| --- \| --- \| \| **Abril** \| **Julho** \| \| **Maio** \| **Agosto** \| | \| **Março** \| **Junho** \| \| --- \| --- \| \| **Abril** \| **Julho** \| \| **Maio** \| **Agosto** \| | \| **Março** \| **Junho** \| \| --- \| --- \| \| **Abril** \| **Julho** \| \| **Maio** \| **Agosto** \| |
| **Videolaparoscopia** | \| **Março** \| **Junho** \| \| --- \| --- \| \| **Abril** \| **Julho** \| \| **Maio** \| **Agosto** \| | \| **Março** \| **Junho** \| \| --- \| --- \| \| **Abril** \| **Julho** \| \| **Maio** \| **Agosto** \| | \| **Março** \| **Junho** \| \| --- \| --- \| \| **Abril** \| **Julho** \| \| **Maio** \| **Agosto** \| | \| **Março** \| **Junho** \| \| --- \| --- \| \| **Abril** \| **Julho** \| \| **Maio** \| **Agosto** \| |
| **Doenças infecciosas trato genital inferior** | \| **Março** \| **Junho** \| \| --- \| --- \| \| **Abril** \| **Julho** \| \| **Maio** \| **Agosto** \| | \| **Março** \| **Junho** \| \| --- \| --- \| \| **Abril** \| **Julho** \| \| **Maio** \| **Agosto** \| | \| **Março** \| **Junho** \| \| --- \| --- \| \| **Abril** \| **Julho** \| \| **Maio** \| **Agosto** \| | \| **Março** \| **Junho** \| \| --- \| --- \| \| **Abril** \| **Julho** \| \| **Maio** \| **Agosto** \| |
| **Uroginecologia** | \| **Março** \| **Junho** \| \| --- \| --- \| \| **Abril** \| **Julho** \| \| **Maio** \| **Agosto** \| | \| **Março** \| **Junho** \| \| --- \| --- \| \| **Abril** \| **Julho** \| \| **Maio** \| **Agosto** \| | \| **Março** \| **Junho** \| \| --- \| --- \| \| **Abril** \| **Julho** \| \| **Maio** \| **Agosto** \| | \| **Março** \| **Junho** \| \| --- \| --- \| \| **Abril** \| **Julho** \| \| **Maio** \| **Agosto** \| |
| **Anticoncepção** | \| **Março** \| **Junho** \| \| --- \| --- \| \| **Abril** \| **Julho** \| \| **Maio** \| **Agosto** \| | \| **Março** \| **Junho** \| \| --- \| --- \| \| **Abril** \| **Julho** \| \| **Maio** \| **Agosto** \| | \| **Março** \| **Junho** \| \| --- \| --- \| \| **Abril** \| **Julho** \| \| **Maio** \| **Agosto** \| | \| **Março** \| **Junho** \| \| --- \| --- \| \| **Abril** \| **Julho** \| \| **Maio** \| **Agosto** \| |

1. Quanto aos ambulatórios de assistência em obstetrícia: (*marcar apenas um check por linha por mês)*

|  | **Redução da oferta de atendimentos** | **Paralisação na oferta de atendimentos** | **Não houve Modificação na oferta de atendimentos** | **Não se aplica (não há esse serviço no centro** |
| --- | --- | --- | --- | --- |
| **Pré-natal baixo risco** | \| **Março** \| **Junho** \| \| --- \| --- \| \| **Abril** \| **Julho** \| \| **Maio** \| **Agosto** \| | \| **Março** \| **Junho** \| \| --- \| --- \| \| **Abril** \| **Julho** \| \| **Maio** \| **Agosto** \| | \| **Março** \| **Junho** \| \| --- \| --- \| \| **Abril** \| **Julho** \| \| **Maio** \| **Agosto** \| | \| **Março** \| **Junho** \| \| --- \| --- \| \| **Abril** \| **Julho** \| \| **Maio** \| **Agosto** \| |
| **Pré-natal alto risco** | \| **Março** \| **Junho** \| \| --- \| --- \| \| **Abril** \| **Julho** \| \| **Maio** \| **Agosto** \| | \| **Março** \| **Junho** \| \| --- \| --- \| \| **Abril** \| **Julho** \| \| **Maio** \| **Agosto** \| | \| **Março** \| **Junho** \| \| --- \| --- \| \| **Abril** \| **Julho** \| \| **Maio** \| **Agosto** \| | \| **Março** \| **Junho** \| \| --- \| --- \| \| **Abril** \| **Julho** \| \| **Maio** \| **Agosto** \| |
| **Ambulatório de consulta puerperal** | \| **Março** \| **Junho** \| \| --- \| --- \| \| **Abril** \| **Julho** \| \| **Maio** \| **Agosto** \| | \| **Março** \| **Junho** \| \| --- \| --- \| \| **Abril** \| **Julho** \| \| **Maio** \| **Agosto** \| | \| **Março** \| **Junho** \| \| --- \| --- \| \| **Abril** \| **Julho** \| \| **Maio** \| **Agosto** \| | \| **Março** \| **Junho** \| \| --- \| --- \| \| **Abril** \| **Julho** \| \| **Maio** \| **Agosto** \| |
| **Serviço de imagem em obstetrícia (Ultrassom, etc)** | \| **Março** \| **Junho** \| \| --- \| --- \| \| **Abril** \| **Julho** \| \| **Maio** \| **Agosto** \| | \| **Março** \| **Junho** \| \| --- \| --- \| \| **Abril** \| **Julho** \| \| **Maio** \| **Agosto** \| | \| **Março** \| **Junho** \| \| --- \| --- \| \| **Abril** \| **Julho** \| \| **Maio** \| **Agosto** \| | \| **Março** \| **Junho** \| \| --- \| --- \| \| **Abril** \| **Julho** \| \| **Maio** \| **Agosto** \| |
| **Laboratório de análises clínicas** | \| **Março** \| **Junho** \| \| --- \| --- \| \| **Abril** \| **Julho** \| \| **Maio** \| **Agosto** \| | \| **Março** \| **Junho** \| \| --- \| --- \| \| **Abril** \| **Julho** \| \| **Maio** \| **Agosto** \| | \| **Março** \| **Junho** \| \| --- \| --- \| \| **Abril** \| **Julho** \| \| **Maio** \| **Agosto** \| | \| **Março** \| **Junho** \| \| --- \| --- \| \| **Abril** \| **Julho** \| \| **Maio** \| **Agosto** \| |

1. Como você considera a adequação sobre os tópicos a seguir? No seu centro e em cada mês específico.

|  | **Suficiente para todas as unidades** | **Suficiente para a maioria das unidades** | **Insuficiente para a maioria das unidades** | **Insuficiente para todas as unidades** |
| --- | --- | --- | --- | --- |
| **Provisão de EPI’s (equipamento de proteção individual)** | \| **Março** \| **Junho** \| \| --- \| --- \| \| **Abril** \| **Julho** \| \| **Maio** \| **Agosto** \| | \| **Março** \| **Junho** \| \| --- \| --- \| \| **Abril** \| **Julho** \| \| **Maio** \| **Agosto** \| | \| **Março** \| **Junho** \| \| --- \| --- \| \| **Abril** \| **Julho** \| \| **Maio** \| **Agosto** \| | \| **Março** \| **Junho** \| \| --- \| --- \| \| **Abril** \| **Julho** \| \| **Maio** \| **Agosto** \| |
| **Treinamento das equipes** | \| **Março** \| **Junho** \| \| --- \| --- \| \| **Abril** \| **Julho** \| \| **Maio** \| **Agosto** \| | \| **Março** \| **Junho** \| \| --- \| --- \| \| **Abril** \| **Julho** \| \| **Maio** \| **Agosto** \| | \| **Março** \| **Junho** \| \| --- \| --- \| \| **Abril** \| **Julho** \| \| **Maio** \| **Agosto** \| | \| **Março** \| **Junho** \| \| --- \| --- \| \| **Abril** \| **Julho** \| \| **Maio** \| **Agosto** \| |
| **Material de apoio para orientar fluxos, decisões e procedimentos.** | \| **Março** \| **Junho** \| \| --- \| --- \| \| **Abril** \| **Julho** \| \| **Maio** \| **Agosto** \| | \| **Março** \| **Junho** \| \| --- \| --- \| \| **Abril** \| **Julho** \| \| **Maio** \| **Agosto** \| | \| **Março** \| **Junho** \| \| --- \| --- \| \| **Abril** \| **Julho** \| \| **Maio** \| **Agosto** \| | \| **Março** \| **Junho** \| \| --- \| --- \| \| **Abril** \| **Julho** \| \| **Maio** \| **Agosto** \| |

1. De forma geral, a entrada de acompanhantes durante a assistência ao parto e no cuidado com o recém-nascido era: (marcar apenas um por linha por mês)

|  | **Sempre** | **Muitas vezes** | **Às vezes** | **Raramente** | **Nunca** |
| --- | --- | --- | --- | --- | --- |
| **No pré-parto** | \| **Março** \| **Junho** \| \| --- \| --- \| \| **Abril** \| **Julho** \| \| **Maio** \| **Agosto** \| | \| **Março** \| **Junho** \| \| --- \| --- \| \| **Abril** \| **Julho** \| \| **Maio** \| **Agosto** \| | \| **Março** \| **Junho** \| \| --- \| --- \| \| **Abril** \| **Julho** \| \| **Maio** \| **Agosto** \| | \| **Março** \| **Junho** \| \| --- \| --- \| \| **Abril** \| **Julho** \| \| **Maio** \| **Agosto** \| | \| **Março** \| **Junho** \| \| --- \| --- \| \| **Abril** \| **Julho** \| \| **Maio** \| **Agosto** \| |
| **No momento do parto** | \| **Março** \| **Junho** \| \| --- \| --- \| \| **Abril** \| **Julho** \| \| **Maio** \| **Agosto** \| | \| **Março** \| **Junho** \| \| --- \| --- \| \| **Abril** \| **Julho** \| \| **Maio** \| **Agosto** \| | \| **Março** \| **Junho** \| \| --- \| --- \| \| **Abril** \| **Julho** \| \| **Maio** \| **Agosto** \| | \| **Março** \| **Junho** \| \| --- \| --- \| \| **Abril** \| **Julho** \| \| **Maio** \| **Agosto** \| | \| **Março** \| **Junho** \| \| --- \| --- \| \| **Abril** \| **Julho** \| \| **Maio** \| **Agosto** \| |
| **No Alojamento conjunto** | \| **Março** \| **Junho** \| \| --- \| --- \| \| **Abril** \| **Julho** \| \| **Maio** \| **Agosto** \| | \| **Março** \| **Junho** \| \| --- \| --- \| \| **Abril** \| **Julho** \| \| **Maio** \| **Agosto** \| | \| **Março** \| **Junho** \| \| --- \| --- \| \| **Abril** \| **Julho** \| \| **Maio** \| **Agosto** \| | \| **Março** \| **Junho** \| \| --- \| --- \| \| **Abril** \| **Julho** \| \| **Maio** \| **Agosto** \| | \| **Março** \| **Junho** \| \| --- \| --- \| \| **Abril** \| **Julho** \| \| **Maio** \| **Agosto** \| |
| **Na UTI neonatal** | \| **Março** \| **Junho** \| \| --- \| --- \| \| **Abril** \| **Julho** \| \| **Maio** \| **Agosto** \| | \| **Março** \| **Junho** \| \| --- \| --- \| \| **Abril** \| **Julho** \| \| **Maio** \| **Agosto** \| | \| **Março** \| **Junho** \| \| --- \| --- \| \| **Abril** \| **Julho** \| \| **Maio** \| **Agosto** \| | \| **Março** \| **Junho** \| \| --- \| --- \| \| **Abril** \| **Julho** \| \| **Maio** \| **Agosto** \| | \| **Março** \| **Junho** \| \| --- \| --- \| \| **Abril** \| **Julho** \| \| **Maio** \| **Agosto** \| |

1. Os exames para confirmar infecção pelo vírus SARS-CoV2 eram: (*marque todas que se aplicam em cada mês)*

***Março***

Realizados no Laboratório do Hospital

Realizados em Laboratório parceiro do Hospital (serviço externo que habitualmente presta serviço ao Hospital)

Instituto Adolfo Lutz (Laboratório nacional)

Laboratório Estadual

Laboratório Municipal

***Abril***

Realizados no Laboratório do Hospital

Realizados em Laboratório parceiro do Hospital (serviço externo que habitualmente presta serviço ao Hospital)

Instituto Adolfo Lutz (Laboratório nacional)

Laboratório Estadual

Laboratório Municipal

***Maio***

Realizados no Laboratório do Hospital

Realizados em Laboratório parceiro do Hospital (serviço externo que habitualmente presta serviço ao Hospital)

Instituto Adolfo Lutz (Laboratório nacional)

Laboratório Estadual

Laboratório Municipal

***Junho***

Realizados no Laboratório do Hospital

Realizados em Laboratório parceiro do Hospital (serviço externo que habitualmente presta serviço ao Hospital)

Instituto Adolfo Lutz (Laboratório nacional)

Laboratório Estadual

Laboratório Municipal

***Julho***

Realizados no Laboratório do Hospital

Realizados em Laboratório parceiro do Hospital (serviço externo que habitualmente presta serviço ao Hospital)

Instituto Adolfo Lutz (Laboratório nacional)

Laboratório Estadual

Laboratório Municipal

***Agosto***

Realizados no Laboratório do Hospital

Realizados em Laboratório parceiro do Hospital (serviço externo que habitualmente presta serviço ao Hospital)

Instituto Adolfo Lutz (Laboratório nacional)

Laboratório Estadual

Laboratório Municipal

1. Quanto tempo em média costumava demorar o resultado do exame? (*em dias*)

- Março:

- Abril:

- Maio:

- Junho:

- Julho:

- Agosto:

1. Quais estratégias estavam em vigor para acompanhar os casos de mulheres sintomáticas e/ou positivas para COVID-19: (*marque todas que se aplicam em cada mês)*

***Março***

Contato por Telefone (ligações)

Contato por mensagens (p.e. WhatsApp)

Vídeochamada

Ambulatório no mesmo serviço

Não havia nenhuma estratégia

Outra – Especifique:

***Abril***

Contato por Telefone (ligações)

Contato por mensagens (p.e. WhatsApp)

Vídeochamada

Ambulatório no mesmo serviço

Não havia nenhuma estratégia

Outra – Especifique:

***Maio***

Contato por Telefone (ligações)

Contato por mensagens (p.e. WhatsApp)

Vídeochamada

Ambulatório no mesmo serviço

Não havia nenhuma estratégia

Outra – Especifique:

***Junho***

Contato por Telefone (ligações)

Contato por mensagens (p.e. WhatsApp)

Vídeochamada

Ambulatório no mesmo serviço

Não havia nenhuma estratégia

Outra – Especifique:

***Julho***

Contato por Telefone (ligações)

Contato por mensagens (p.e. WhatsApp)

Vídeochamada

Ambulatório no mesmo serviço

Não havia nenhuma estratégia

Outra – Especifique:

***Agosto***

Contato por Telefone (ligações)

Contato por mensagens (p.e. WhatsApp)

Vídeochamada

Ambulatório no mesmo serviço

Não havia nenhuma estratégia

Outra – Especifique:
