# Supplementary material for: Facing the COVID-19 pandemic inside maternities in Brazil: A mixed-method study within the REBRACO initiative
Source: PLoS One. 2021 Jul 23;16(7):e0254977. doi: 10.1371/journal.pone.0254977 (PMC8301675; doi:10.1371/journal.pone.0254977)
Supplement: S3 Data — Semi-structured interview script for health managers, used on the qualitative study. (DOCX) [file pone.0254977.s004.docx]

**Supporting information S3**: Semi-structured interview script for health managers, used on the qualitative study

**Semi-structured interview script - English**

**Characteristics of the health manager:**

1. What’s your gender?

2. How old are you?

3. What’s your professional qualification?

4. What’s your position? And your degree?

5. What’s your role in the institution?

6. For how many years have you worked at the institution?

7. Where is your institution?

**Semi-structured interview script**

1. Could you tell us about how you managed the preparation for facing the pandemic in your institution?

2. How was your experience in developing protocols and workflows based on the great number of sources of information (WHO, MoH, etc) and frequent updates?

3. How was the acceptance by the health professionals regarding the workflow and protocols established by the institution? What about training programs?

4. Has any educational program about COVID-19 focused on the women or the general audience been launched in the maternity?

5. Could you please tell u show was the experience to deal with the first suspected or positive COVID-19 cases in your unit and the related challenges at that moment?

6. In your opinion, how stressful the nursing and medical teams were? How was it to deal with this situation?

7. Did the institution develop any mental health program for health professionals? Has the institution had health professional on leave due to stress?

8. How is your stress level? How has your experience on dealing with the pandemic at work affected you personnel and professional life?

9. How did you feel about the suspected or confirmed diagnosis?

10. Considering all actions implemented in the response program, which ones do you consider to have been positive in relation to health professional, users and to the institution itself?

11. What was the main challenge that you faced as a health manager when implementing the response program? (e.g. economic restraints, lack of health professionals, lack of resources such as tests or personnel protective equipment).

12. How did you, as health manager, feel about the challenge of organizing the response to the pandemic in your maternity/hospital?

13. Would you like to add anything else?

**Semi-structured interview script – Portuguese (original)**

**Características do gestor:**

1. Qual seu sexo? Gênero?

2. Quantos anos completos tem?

3. Qual é a sua formação profissional?

4. Qual o último grau de escolaridade que completou?

5. Qual(is) função (ões) exerce na instituição?

6. Há quantos anos é gestor dessa instituição?

7. Em qual cidade a instituição está localizada?

**Roteiro entrevista semiestruturada**

1. Gostaria que você me contasse como você, como gestor, se mobilizou para preparar a maternidade para enfrentar a pandemia?

2. Como foi sua experiência em ter que elaborar protocolos e fluxogramas com uma quantidade enorme de informações oficiais (OMS, Ministério da Saúde) que se alteravam frequentemente?

3. Como foi a aceitação pelos profissionais de saúde quanto aos fluxogramas e protocolos estabelecidos na instituição? E quanto aos treinamentos?

4. Foi feita alguma ação educativa sobre a Covid-19 direcionada para as mulheres/público em geral que frequenta sua maternidade?

5. Gostaria que você me contasse como foi quando os primeiros casos suspeitos de Covid-19 ou positivos começaram a chegar na maternidade e os desafios que enfrentaram nesse momento?

6. Na sua opinião como está o nível se estresse das equipes de enfermagem e médicas? Como foi lidar com essa situação?

7. A instituição elaborou algum atendimento à saúde mental dos profissionais de saúde? Teve funcionários que foram afastados por stress?

8. Como está o seu nível de stress? A vivência que você teve no trabalho quanto ao enfrentamento a pandemia de que forma afetou sua vida pessoal e profissional?

9. Você contraiu a doença? Como se sentiu diante do diagnóstico confirmado ou de suspeita?

10. De todas as ações realizadas para o enfrentamento da pandemia, quais considera que efetivamente foram positivas em relação aos profissionais de saúde, frequentadores da maternidade e também para a própria instituição?

11. Qual foi o maior desafio que você enfrentou como gestor para implementar o programa de enfrentamento durante a pandemia? (restrições econômicas, poucos profissionais, falta de recursos específicos como testes, material de proteção etc)

12. Como você, como gestor, se sentiu diante desse desafio de ter que organizar a maternidade/hospital para enfrentar a pandemia?

13.Gostaria de falar mais alguma coisa sobre esse assunto?
