# Supplementary material for: Facing the COVID-19 pandemic inside maternities in Brazil: A mixed-method study within the REBRACO initiative
Source: PLoS One. 2021 Jul 23;16(7):e0254977. doi: 10.1371/journal.pone.0254977 (PMC8301675; doi:10.1371/journal.pone.0254977)
Supplement: Attachment — Submitted filename: Response_PloseOne_R1_.docx [file pone.0254977.s005.docx]

**Point-by-Point Responses**

Dear editor,

We acknowledge the detailed evaluation of our study; points raised by the reviewers were important and helped to improve our manuscript.

Bellow, you will find a point-by-point response answering reviewer’s comments. All changes are highlighted (in yellow) in the manuscript and cover letter.

Sincerely,

Maria Laura Costa, MD, PhD

**1. Please ensure that your manuscript meets PLOS ONE's style requirements, including those for file naming. The PLOS ONE style templates can be found at**

<https://journals.plos.org/plosone/s/file?id=wjVg/PLOSOne_formatting_sample_main_body.pdf> and

<https://journals.plos.org/plosone/s/file?id=ba62/PLOSOne_formatting_sample_title_authors_affiliations.pdf>

**RESPONSE:** Thank you. We have checked the style requirements and made the needed modifications.

2. Thank you for including your ethics statement: "Ethical approval for the main study and in each participating center was obtained (Letter of Approval numbers 4.047.168, 4.179.679, and 4.083.988)."   
 a.Please amend your current ethics statement to include the full name of the ethics committee/institutional review board(s) that approved your specific study.
 b.Once you have amended this/these statement(s) in the Methods section of the manuscript, please add the same text to the “Ethics Statement” field of the submission form (via “Edit Submission”).
 For additional information about PLOS ONE ethical requirements for human subjects research, please refer to <http://journals.plos.org/plosone/s/submission-guidelines#loc-human-subjects-research>.

**RESPONSE:** The full name of the ethics committee/institutional review boards that approved the study were included- page 3-4:

“Ethical approval for the main study and in each participating center was obtained (Letter of Approval numbers 4.047.168, 4.179.679, and 4.083.988, from the coordinating center´s Institutional Review Board from the University of Campinas, the IRB of the School of Medicine of Jundiai, IRB of the Clinics Hospital of Porto Alegre, the IRB of the Unimed Maternity of Belo Horizonte, the IRB of MEAC from the Federal University of Ceara, the IRB of the Federal University of Sao Paulo, IRB of Moinhos de Vento Hospital from Porto Alegre, IRB of the Jorge Rossmann Regional Hospital from Itanhaem, IRB of the Federal University of Sao Carlos, IRB of the State Hospital of Sumare, IR of the Feral University of Minas Gerais, IRB of the Fernandes Figueira Institute from Fiocruz in Rio de Janeiro, IRB of the School of Medicine from the State University of Sao Paulo in Botucatu, IRB of the Federal University of Pernambuco, IRB of the Climerio de Oliveira Maternity from the Federal University of Bahia in Salvador, and the IRB of the Santa Casa de Misericordia do Pará from Belem.”

**3. For your cross-sectional study, please provide additional details regarding participant consent. In the ethics statement in the Methods and online submission information, please ensure that you have specified (1) whether consent was obtained for the questionnaire (2) whether consent was informed and (2) what type you obtained (for instance, written or verbal, and if verbal, how it was documented and witnessed).  If the need for consent was waived, please ensure that you have discussed whether all data were fully anonymized before you accessed them and/or whether the IRB or ethics committee waived the requirement for informed consent.**

**4. Regarding verbal consent in your qualitative study, in the Methods, please state:
- Why written consent could not be obtained
- Whether the Institutional Review Board (IRB) approved use of oral consent
- How oral consent was documented
For more information, please see our guidelines for human subjects research:** <https://journals.plos.org/plosone/s/submission-guidelines#loc-human-subjects-research>

**RESPONSE: Considering questions 3 and 4-** Thank you for your comment and questions. This manuscript represents a great study initiative in Brazil, called REBRACO, with many aims to broadly understand the impact of the COVID-19 in our scenario. Therefore, we have split this major project in three, for ethical approval, dividing the cross-sectional, cohort of suspected cases and qualitative approaches. That is why we presented 3 ethical approval numbers above. We have authorization for both- written informed consent and consent by phone, with authorized recording. Mostly suspected cases with hospital admissions have written informed consent, cases that were evaluated and tested in the emergency room, with no needed admission were later consented by phone. Qualitative studies, especially the one presented here, with health professionals, were done remotely, by phone, since maternities considered are in different parts of the country and all interviews were performed by the same researcher in the coordinating center.

We would like to acknowledge the fact that the current study was based on aggregated data collected via the data collection form that was made available as supporting information and that individual data related to the number of suspected and/or confirmed COVID-19 cases and the related clinical characterization, rate of progression to acute respiratory distress syndrome, and mortality will be properly and timely addressed in another publication of the Consortium.

The information is now clearer in the manuscript, methods, page 5:

“All suspected cases of COVID-19 in the considered maternities were assessed for informed consent and their data was only included after such consent. The study has authorization for both- written informed consent and consent by phone to use information from medical records and sample collection. Mostly suspected cases with hospital admissions have written informed consent, cases that were evaluated and tested in the emergency room, with no needed admission were later consented by phone. The researcher read and explained the consent form and assured that women could withdraw from the study without any interference with planned medical care. All interviews were recorded on audio after consent from the participants, using the Microsoft Skype platform.

The data sharing information on the questionnaire of readiness towards the pandemic and overall numbers of each institution was approved by the coordinating center and locally in each participating center. Data was stored in a specific database created for this study and protected by password in a de-identified way. Considering biosafety reasons, informed consent by telephone contact was approved by the Institutional Review Board. Participant identity and all personal data as well will remain confidential.

We also performed a qualitative component, considering key obstetricians involved in the decision making of each institution, to listen to their perspectives on the challenges to face the pandemic in their setting. These participants were identified by local PIs (principal investigators). A qualitative study by purposeful sampling and saturation sampling was undertaken after individual oral consent. All semi-structured interviews were performed by a skilled social researcher with experience in the field and were recorded on audio. For this approach, the Institutional Review Board (IRB) approved the use of oral consent. Maternities considered are in different regions of the country and all interviews were performed by the same researcher in the coordinating center, by telephone, with specific software for further transcription and analysis (“ReShape”), protected by password in a de-identified manner.”

**5. In your Methods section, please provide additional information about the participant recruitment method and the demographic details of your participants. Please ensure you have provided sufficient details to replicate the analyses such as: a) the recruitment date range (month and year), b) a description of any inclusion/exclusion criteria that were applied to participant recruitment, c) a table of relevant demographic details, d) a statement as to whether your sample can be considered representative of a larger population.**

**RESPONSE:** thank you for your comment, we understand that it was not clear that we here present part of a major study called REBRACO. We have included a paragraph to ascertain that this is now accurate- page 3

“The REBRACO study aims to broadly understand the impact of the COVID-19 in the considered institutions, evaluating clinical, epidemiological and laboratory aspects of SARS-CoV-2 infection during pregnancy and the postpartum (11). The current analysis presents data on how centers faced the pandemic and prepared each institution for the identification and medical care of patients, considering information from January to July 31, 2020.”

Considering the selection of cases for the qualitative component of the current analysis, the local PI identified key health professionals/obstetricians involved in the decision making of each institution and/or responsible for the maternities’ response towards the pandemic. The phone contact to obtain ethical informed consent and the interview were performed by the same research assistant, from the coordinating center. We have added a sentence to the paragraph in page 5:

“We also performed a qualitative component, considering key obstetricians involved in the decision making of each institution, to listen to their perspectives on the challenges to face the pandemic in their setting. These participants were identified by local PIs (principal investigators).”

The details on the cohort of suspected/positive COVID-19 cases, with demographic, clinical, maternal and perinatal outcomes will be further presented in a future analysis. We have included in the discussion, relevant information about Brazil, with data from published studies (from National surveillance system) analyzing clinical characteristics and risk factors for COVID-19-related maternal deaths in the country, that showed the postpartum period, obesity, diabetes, cardiovascular disease and black ethnicity as conditions associated with the fatality rate.

The REBRACO initiative includes 16 maternity hospitals, in 4 of the five regions of the country, mostly University Hospitals, with intensive care support and referral centers for maternal healthcare. Therefore, we understand that it is not representative of the country. Brazil is a country of continental size and most of our centers concentrate in the southeast region. We have included a paragraph in the discussion to add this information.

“The institutions considered in this study, mostly university hospitals and referral centers, had availability for such support, what might not be true or representative of most maternities in Brazil.”

**6. Please include a copy of the interview guide used in the qualitative study, in both the original language and English, as Supporting Information, or include a citation if it has been published previously**.

**RESPONSE:** Thank you for the suggestion. We included the semi-structured interview script in both languages (Portuguese and English) as supporting information (S2). The respective reference to the document was included in the methods (Page 4-5).

7**. Please include additional information regarding the survey or questionnaire used in the cross-sectional study and ensure that you have provided sufficient details that others could replicate the analyses. For instance, if you developed a questionnaire as part of this study and it is not under a copyright more restrictive than CC-BY, please include a copy, in both the original language and English, as Supporting Information.**

**RESPONSE:** Thank you for your suggestion. In addition to the detailed description of variables included in the data collection form, we included the data collection form that was sent to the health managers as Supporting Information. This information was included in the methods (Page 4-5).

“The data collection form is provided as Supporting Information (S1). Data were collected with the assistance of the maternity hospital staff. We conducted a descriptive analysis of the information above, including the characteristics of the maternities, their response program, the training program and some indicators related to the COVID-19 pandemic such as the number of cases, number of maternal deaths, maternal mortality ratio due to COVID-19 in the period, time interval between the implementation of the response program and the first suspected COVID-19 cases in each center and the characteristics of the tests available in the obstetric units.”

**8. Please include a discussion of any limitations to your study in the Discussion section.**

**RESPONSE:** We have included a paragraph in the discussion, with the main identified limitations of the study. Page 21:

“As a limitation of the current REBRACO study, data from the 16 maternity centers included is not representative of the real maternal health care throughout Brazil, a country of continental size and major disparities. Therefore, the presented results do not provide data to ascertain improved clinical outcomes among considered maternities. In addition, as previously informed, the centers did not follow a common standard protocol for clinically dealing with cases of Covid19 during pregnancy, because only very recently the Ministry of Health issued general recommendations for such management.”

**To comply with PLOS ONE submission guidelines, in your Methods section, please provide additional information regarding your statistical analyses. For more information on PLOS ONE's expectations for statistical reporting, please see** <https://journals.plos.org/plosone/s/submission-guidelines.#loc-statistical-reporting>.

**RESPONSE:** Thank you for the suggestion. The following information was added to the manuscript in the methods (Page 4-5):

“We conducted a descriptive analysis of the information above, including the characteristics of the maternities, their response program, the training program and some indicators related to the COVID-19 pandemic such as the number of cases, number of maternal deaths, maternal mortality ratio due to COVID-19 in the period, time interval between the implementation of the response program and the first suspected COVID-19 cases in each center and the characteristics of the tests available in the obstetric units.”

“Thematic analysis was finally carried out [12] after the familiarization with the collected data. Then we identified the main themes after generating the initial codes. The themes were reviewed and, finally, they were defined and named according to the main challenges identified in the interviewees’ narratives, with their corresponding experiences, conflicts and answers/actions.”

We would like to acknowledge the fact that we only conducted descriptive and thematic analyses for the quantitative and qualitative components of the study, respectively.

**10.Thank you for stating the following in the Acknowledgments Section of your manuscript:**

**"The study was supported by FAEPEX-Unicamp (Fundo de Apoio ao Ensino, à Pesquisa e à Extensão) under the grant number 2300/20."**

**We note that you have provided funding information that is not currently declared in your Funding Statement. However, funding information should not appear in the Acknowledgments section or other areas of your manuscript. We will only publish funding information present in the Funding Statement section of the online submission form.**

**Please remove any funding-related text from the manuscript and let us know how you would like to update your Funding Statement. Currently, your Funding Statement reads as follows:**

**"The funders had no role in study design, data collection and analysis, decision to publish, or preparation of the manuscript."**

**Please include your amended statements within your cover letter; we will change the online submission form on your behalf.**

**RESPONSE:** Thank you. We have now corrected the above text and removed funding-related information from acknowledgements and included the amended statements within our cover letter.

**11. Please amend either the title on the online submission form (via Edit Submission) or the title in the manuscript so that they are identical.**

**RESPONSE:** We are sorry. The title was corrected in the submission.

12. One of the noted authors is a group or consortium REBRACO Study Group#. In addition to naming the author group, please list the individual authors and affiliations within this group in the acknowledgments section of your manuscript. Please also indicate clearly a lead author for this group along with a contact email address.

**RESPONSE:** The lead author for this group is Jose Guilherme Cecatti, the last author of this manuscript.

The individual authors and affiliation and the indication of the lead author for each group with a contact e-email address within the respective group were added in the acknowledgments accordingly as suggested.

**13.We note that [Figure(s) 1] in your submission contain map images which may be copyrighted. All PLOS content is published under the Creative Commons Attribution License (CC BY 4.0), which means that the manuscript, images, and Supporting Information files will be freely available online, and any third party is permitted to access, download, copy, distribute, and use these materials in any way, even commercially, with proper attribution. For these reasons, we cannot publish previously copyrighted maps or satellite images created using proprietary data, such as Google software (Google Maps, Street View, and Earth). For more information, see our copyright guidelines:** [**http://journals.plos.org/plosone/s/licenses-and-copyright**](http://journals.plos.org/plosone/s/licenses-and-copyright)**.**

**We require you to either (1) present written permission from the copyright holder to publish these figures specifically under the CC BY 4.0 license, or (2) remove the figures from your submission:**

**a.    You may seek permission from the original copyright holder of Figure(s) [1] to publish the content specifically under the CC BY 4.0 license.**

**We recommend that you contact the original copyright holder with the Content Permission Form (**[**http://journals.plos.org/plosone/s/file?id=7c09/content-permission-form.pdf**](http://journals.plos.org/plosone/s/file?id=7c09/content-permission-form.pdf)**) and the following text:**

**“I request permission for the open-access journal PLOS ONE to publish XXX under the Creative Commons Attribution License (CCAL) CC BY 4.0 (**[**http://creativecommons.org/licenses/by/4.0/**](http://creativecommons.org/licenses/by/4.0/)**). Please be aware that this license allows unrestricted use and distribution, even commercially, by third parties. Please reply and provide explicit written permission to publish XXX under a CC BY license and complete the attached form.”**

**Please upload the completed Content Permission Form or other proof of granted permissions as an "Other" file with your submission.**

**In the figure caption of the copyrighted figure, please include the following text: “Reprinted from [ref] under a CC BY license, with permission from [name of publisher], original copyright [original copyright year].”**

**b.    If you are unable to obtain permission from the original copyright holder to publish these figures under the CC BY 4.0 license or if the copyright holder’s requirements are incompatible with the CC BY 4.0 license, please either i) remove the figure or ii) supply a replacement figure that complies with the CC BY 4.0 license. Please check copyright information on all replacement figures and update the figure caption with source information. If applicable, please specify in the figure caption text when a figure is similar but not identical to the original image and is therefore for illustrative purposes only.**

**The following resources for replacing copyrighted map figures may be helpful:**

USGS National Map Viewer (public domain): <http://viewer.nationalmap.gov/viewer/>

The Gateway to Astronaut Photography of Earth (public domain): <http://eol.jsc.nasa.gov/sseop/clickmap/>

Maps at the CIA (public domain): <https://www.cia.gov/library/publications/the-world-factbook/index.html> and <https://www.cia.gov/library/publications/cia-maps-publications/index.html>

NASA Earth Observatory (public domain): <http://earthobservatory.nasa.gov/>

Landsat: <http://landsat.visibleearth.nasa.gov/>

USGS EROS (Earth Resources Observatory and Science (EROS) Center) (public domain): [http://eros.usgs.gov/#](http://eros.usgs.gov/)

Natural Earth (public domain): <http://www.naturalearthdata.com/>

**RESPONSE:** Figure 1 was adapted from a map contained in the Brazilian public domain database – *Portal Domínio Público* (<http://www.dominiopublico.gov.br>). Thus, the image has no copyright linked to be evidenced. We have included this information in the Figure footnote

**Additional Editor Comments (if provided):

Please address carefully the points below:
1) Clarify (abstract and method section) the study design: is this an observational mixed method study? was there an intervention component?**

**RESPONSE:** Thank you. This is an observational mixed method study. We have made that clear in the abstract and methods. There were no interventions.

**2) Move the description of the REBRACO initiative (lines 381- 404) in the method section, clarifying to which extent there was an intervention, and when. Do separate activities which related to the study period from activities which has been undertaken after this study**

**RESPONSE:** Thank you. This paragraph is important to add information on the activities of the REBRACO study group. The content of these meetings have inspired the current manuscript, as we learned with each of the local center´s presenting their challenges and ways to overcome them. We have summarized the paragraph and made it clear that there were no interventions. It is now the last paragraph of the methods.

“In the REBRACO initiative, there are no planned interventions in each included centers. All the research team, including local-PIs, health professionals involved in the local Emergency Action Committees, and other collaborators (Ob&Gyn residents, consultants, etc.) from all the included centers, have been invited to participate in weekly virtual meetings, sharing information regarding the organization of the different health services, the barriers and facilitators in the implementation of healthcare and training of health professionals in each center and discussions on current findings on maternal and perinatal outcomes during pregnancy and postpartum of COVID-19 infected women. These meetings have inspired the current analysis.”

3) Refer to the appropriate guidelines of reporting based on the study design (see <https://www.equator-network.org/reporting-guidelines/>)

**RESPONSE:** STROBE guideline was followed and included as a supplementary material.

4) Consider revising the title based on the study design and findings, ie, this specific manuscript does not seem to directly aim at exploring how to overcome challenges to adequate care (This would require an intervention study), neither it shows that challenges has been overcomed (table 2) - rather it seems to provide an analysis of the challenges faced by hospitals in a research network and related indicators

**RESPONSE:** Thank you. You are correct, we have changed the title according to the above suggestion. It now reads: “**Facing the COVID-19 Pandemic inside maternities in Brazil: challenges towards adequate care in the REBRACO initiative”**

5) Clarify the case definition for "MD due to COVID", and, if possible, add details on case characteristics. Adequately comment in the discussion section: how this MD rate compare to the usual MD rates in the included hospitals? where cases clustered or equally distributed among hospitals? where MD most probably due to COVID our to low quality of care?

**RESPONSE:** Thank you for the comment and questions. The focus of the current analysis is based on the topics presented. We intend to further detail maternal deaths due to COVID in another analysis and that is why we did not present data specific to reported cases, such as sociodemographic information, clinical background, maternal and perinatal outcomes. We here presented overall numbers, to provide a better picture of the impact of the pandemic. There were 15 maternal deaths due to COVID-19 in the participating centers during the study period. The 29 maternal deaths were due to all causes (and the main cause in Brazil is hypertension). This is described in page 6. Each participating center presented its data according to the information in medical charts.

“There were overall 29 maternal deaths (distributed in only 7 of the 16 centers) and 15 (51.8%) attributed to COVID-19 (in 6 centers), with a Maternal mortality rate (MMR) of 127.8/100.000 LB.”

The discussion if MD were most probably due to COVID or to low quality of care is very complex and we cannot answer it yet, in this manuscript. However, it is important to consider published information about Brazil, with data from our national surveillance system. Data on maternal mortality due to COVID-19 in Brazil showed that up to July 14, 2020, 2,475 cases of COVID‐19 acute respiratory distress syndrome (ARDS) were notified, and among them 204 maternal deaths occurred. Delays in receiving care may have played an important role in fatality rate due to COVID19 among pregnant/postpartum women in our country. Initial “stay at home” recommendation and restrictions on public transportation lengthened hospital admission, characterizing the first delay. Social vulnerability may have led to delays on reaching ICUs, corresponding to the second delay. Lack of thromboprophylaxis after cesarean in high‐risk pregnant women may be an example of the third delay (Menezes, M.O., Takemoto, M.L.S., Nakamura‐Pereira, M., Katz, L., Amorim, M.M.R., Salgado, H.O., Melo, A., Diniz, C.S.G., de Sousa, L.A.R., Magalhaes, C.G., Knobel, R., Andreucci, C.B. and (2020), Risk factors for adverse outcomes among pregnant and postpartum women with acute respiratory distress syndrome due to COVID‐19 in Brazil. Int. J. Gynecol. Obstet., 151: 415-423). We believe that maternal deaths are related both to the severity of coronavirus disease upon obstetric patients, but also due to inappropriate care, including delays.

However, comparing with nationwide data, we understand that the REBRACO study group does not represent healthcare in the country, with most centers in the southeast region and with better maternal and perinatal outcomes, since most centers are University Hospitals, with availability of Intensive Care Support. However, we have highlighted in the discussion, that most cases of MD (12 deaths)- occurred in the North and Northeast regions (worst HDI- Human Development Index), what corroborates to previous data on worse outcomes in under-resourced settings (page 22)

6) Ensure that all statements in the discussion section have an appropriate reference (eg, line 327 the reference on the Brazilian epidemiological reports is lacking; line 353 the reference open the recent report from Brazil is also missing; line 405 the statement on "major limitation in many settings" miss a reference; please do revise the whole discussion section)

**Response:** The respective references were added and the final list of references was updated. We also revised the discussion section accordingly.

7) Improve the quality of English (eg ask a native English speaker to revise the manuscript)

**Response:** English has been revised.

8) Revise the manuscript according Plos guidelines <https://journals.plos.org/plosone/s/submission-guidelines>


[Note: HTML markup is below. Please do not edit.]

Reviewers' comments:

Reviewer's Responses to Questions

**Comments to the Author**

1. Is the manuscript technically sound, and do the data support the conclusions?

The manuscript must describe a technically sound piece of scientific research with data that supports the conclusions. Experiments must have been conducted rigorously, with appropriate controls, replication, and sample sizes. The conclusions must be drawn appropriately based on the data presented.

Reviewer #1: Yes

Reviewer #2: Partly

2. Has the statistical analysis been performed appropriately and rigorously?

Reviewer #1: I Don't Know

Reviewer #2: N/A

3. Have the authors made all data underlying the findings in their manuscript fully available?

The [PLOS Data policy](http://www.plosone.org/static/policies.action#sharing) requires authors to make all data underlying the findings described in their manuscript fully available without restriction, with rare exception (please refer to the Data Availability Statement in the manuscript PDF file). The data should be provided as part of the manuscript or its supporting information, or deposited to a public repository. For example, in addition to summary statistics, the data points behind means, medians and variance measures should be available. If there are restrictions on publicly sharing data—e.g. participant privacy or use of data from a third party—those must be specified.

Reviewer #1: Yes

Reviewer #2: Yes

4. Is the manuscript presented in an intelligible fashion and written in standard English?

PLOS ONE does not copyedit accepted manuscripts, so the language in submitted articles must be clear, correct, and unambiguous. Any typographical or grammatical errors should be corrected at revision, so please note any specific errors here.

Reviewer #1: Yes

Reviewer #2: Yes

5. Review Comments to the Author

Please use the space provided to explain your answers to the questions above. You may also include additional comments for the author, including concerns about dual publication, research ethics, or publication ethics. (Please upload your review as an attachment if it exceeds 20,000 characters)

Reviewer #1: Good relevant study with such substantial information added on how to deal with pandemic especially in Low-middle and relevant to high income economies.

Few queries:

**Testing challenges; It is mentioned testing was performed full stop; but no further information on how testing was done. For lessons to other parts of the world, it would best to describe how testing was performed. Done by patients themselves or health providers? Inline with testing, any particular lessons we can learn to why there were delays in getting back the results in some centres? It can be questionable if attributable to low resources or fear of sampling and analysis.**

**RESPONSE:** We included further information about the tests that had been performed in the REBRACO centers during the study period. The following sentence and the respective Figure was included in the results (Page 8).

“Figure 2 shows the characteristics of the tests performed in the REBRACO centers. In half of the centers (n=8), the tests were not performed in the facility and the characteristics were not available. The characteristic of the RT-PCR and serological tests available were heterogeneous across centers.”

Page 19 addresses the topics raised by the reviewers.

“A major limitation in many settings, as shown in the current results, is the restricted availability of testing for SARS-CoV-2 and when available, the delayed turnover of results and heterogeneity of the characteristics of the tests across centers.”

“In addition, the heterogeneity of tests make the comparison of the incidence of exposed women to COVID-19 more difficult, which increase challenges on the response to the pandemic using such epidemiological information.”

**Number of cases, challenges and strategies: 29 death attributable to COVD-19; any particular risk factors, co-morbidities ?**

**RESPONSE:** Actually, there were 15 maternal deaths due to COVID-19 in the participating centers during the study period. The 29 maternal deaths were due to all causes (and the main cause in Brazil is hypertension). This is described in page 6:

“There were overall 29 maternal deaths (distributed in only 7 of the 16 centers) and 15 (51.8%) attributed to COVID-19 (in 6 centers), with a Maternal mortality rate (MMR) of 127.8/100.000 LB.”

We would like to clarify that we intend to explore the maternal deaths in detail in another analysis which includes the presence of risk-factors, co-morbidities, management characteristics, etc. We thank you for your understanding. However, we have improved the discussion on the scenario and numbers of maternal deaths due to COVID-19 in Brazil and delays associated to such numbers.

Reviewer #2: While the work of the authors in preparing their centers for the care of pregnant women is laudable and to be commended, I am not sure how this paper, other than by describing the REBRACO program, provides any evidence that such a program provided for improved care and led to reduced maternal/neonatal morbidity or mortality. There are no comparisons to any non-REBRACO participating centers and there isn't even a comparison of the maternal death rate during the pandemic months in the REBRACO centres as compared to, say, the same centers during a similar time period before the pandemic. Many such multicenter programs have been set up world wide to better share scarce resources (i.e., PPE), coordinate referrals, better communicate clinical advances and risks and to best document clinical outcomes. While the authors have well reported the clinical outcomes, there needs to be some determination as to whether this approach actually provided for improved clinical outcomes.

**RESPONSE:** Thank you for this consideration. We understand the title was inadequate and misleading, since there are no interventions reported or analysis to compare outcomes of centers in the REBRACO and out of the research initiative. Our aim was to report challenges on facing the pandemic from centers in a middle-income setting. We presented quantitative and qualitative data to complement the analysis and this is one of the strengths of the paper. At this point, we cannot ascertain that this approach actually provided for improved clinical outcomes, however, we do believe that the participation in a committed group, including health managers, with weekly meetings on the topic and shared experiences can help in overcoming challenges. We have now better explained methods and improved discussion, to make this clear and to point the study limitations.

6. PLOS authors have the option to publish the peer review history of their article ([what does this mean?](https://journals.plos.org/plosone/s/editorial-and-peer-review-process#loc-peer-review-history)). If published, this will include your full peer review and any attached files.


If you choose “no”, your identity will remain anonymous but your review may still be made public.

**Do you want your identity to be public for this peer review?** For information about this choice, including consent withdrawal, please see our [Privacy Policy](https://www.plos.org/privacy-policy).

Reviewer #1: No	Reviewer #2: No

**Point-by-Point Response- after submitted R1 (please consider both responses)**

Dear editor,

Based on the requests bellow, we have updated our previous response. Thank you for the careful evaluation of the manuscript.

Bellow, you will find a point-by-point response answering reviewer’s comments. All changes are highlighted (in yellow) in the manuscript and cover letter.

Sincerely,

Maria Laura Costa, MD, PhD

PONE-D-20-29941R1
Facing the COVID-19 Pandemic inside maternities in Brazil: challenges towards adequate care in the REBRACO initiative
Dr Maria L Costa

Dear Dr Costa,

Thank you for submitting your manuscript entitled "Facing the COVID-19 Pandemic inside maternities in Brazil: challenges towards adequate care in the REBRACO initiative" to PLOS ONE.  Your manuscript files have been checked in-house but before we can proceed we need you to address the following issues:

1) Please remove your figures/ from within your manuscript file, leaving only the individual TIFF/EPS image files.  These will be automatically included in the reviewer’s PDF

**RESPONSE:** Figures removed.

2) Thank you for removing the following from the Acknowledgments Section of your manuscript:
""The study was supported by FAEPEX-Unicamp (Fundo de Apoio ao Ensino, à
Pesquisa e à Extensão) under the grant number 2300/20."

Currently, your Funding Statement reads as follows: "The funders had no role in study design, data collection and analysis, decision to publish, or preparation of the manuscript."
Please provide an amended statement that declares *all* the funding or sources of support (whether external or internal to your organization) received during this study, as detailed online in our guide for authors at <http://journals.plos.org/plosone/s/submit-now>.  Please also include the statement “There was no additional external funding received for this study.” in your updated Funding Statement.
Unfortunately the funding section on the submission form is locked down to authors at revision so any changes would have not been saved, therefore can you please add your financial statement to your cover letter and we will update the submission form on your behalf.

**RESPONSE:** We have included as requested- details are in the cover letter

“The funders had no role in study design, data collection and analysis, decision to publish, or preparation of the manuscript. The funding source of support during this study was FAEPEX-Unicamp (Fundo de Apoio ao Ensino, à Pesquisa e à Extensão) under grant number 2300/20.”

3) "Thank you for your response to our recent requests regarding patient consent. In order to confirm that your manuscript complies with our reporting guidelines for human subject research (<https://journals.plos.org/plosone/s/submission-guidelines#loc-human-subjects-research>), please could you clarify the following points.

1. On line 135, you state "All suspected cases of COVID-19 in the considered maternities were assessed for informed consent and their data was only included after such consent." In the Methods section, please clarify the context in which consent was obtained, and specify whether patients provided:
        a) Consent to use their medical records/samples used in research
        OR b) Consent to take part in the study reported in this manuscript.
In addition, please state whether the type of consent was written.

**RESPONSE:** Thank you. We agree that the explanation provided in the response was not as clear in the manuscript. Details on consent were better explained. We have authorization for both- written informed consent and consent by phone to use information from medical records. Mostly suspected cases with hospital admissions have written informed consent (including consent for biological sample collection), cases that were evaluated and tested in the emergency room, with no needed admission were later consented by phone, considering biosafety issues and challenges in scheduling medical visits. Qualitative studies, especially the one presented here, with health professionals, were done remotely, by phone, since maternities considered are in different parts of the country and all interviews were performed by the same researcher in the coordinating center.

The paragraph in page 4 now reads:

“All suspected cases of COVID-19 in the considered maternities were assessed for informed consent and their data was only included after such consent. The study has authorization for both- written informed consent and consent by phone to use information from medical records and sample collection. Mostly suspected cases with hospital admissions have written informed consent, cases that were evaluated and tested in the emergency room, with no needed admission were later consented by phone. The researcher read and explained the consent form and assured that women could withdraw from the study without any interference with planned medical care. All interviews were recorded on audio after consent from the participants, using the Microsoft Skype plataform.

The data sharing information on the questionnaire of readiness towards the pandemic and overall numbers of each institution was approved by the coordinating center and locally in each participating center. Data was stored in a specific database created for this study and protected by password in a de-identified way. Considering biosafety reasons, informed consent by telephone contact was approved by the Institutional Review Board. Participant identity and all personal data as well will remain confidential.

We also performed a qualitative component, considering key obstetricians involved in the decision making of each institution, to listen to their perspectives on the challenges to face the pandemic in their setting. These participants were identified by local PIs (principal investigators). A qualitative study by purposeful sampling and saturation sampling was undertaken after individual oral consent. All semi-structured interviews were performed by a skilled social researcher with experience in the field and were recorded on audio. For this approach, the Institutional Review Board (IRB) approved the use of oral consent. Maternities considered are in different regions of the country and all interviews were performed by the same researcher in the coordinating center, by telephone, with specific software for further transcription and analysis (“ReShape”), protected by password in a de-identified manner.”

2. On line 140, you state "For mild cases not admitted to the hospital, considering biosafety reasons, informed consent by telephone contact was approved by the Institutional Review Board." In the Methods section, please state how verbal consent was documented/recorded.

**RESPONSE:** verbal consent was recorded using the Microsoft Skype platform. This is now clear in page 5.
