# Supplementary material for: Facing the COVID-19 pandemic inside maternities in Brazil: A mixed-method study within the REBRACO initiative
Source: PLoS One. 2021 Jul 23;16(7):e0254977. doi: 10.1371/journal.pone.0254977 (PMC8301675; doi:10.1371/journal.pone.0254977)
Supplement: Attachment — Submitted filename: Response_PloseOne_R2_.docx [file pone.0254977.s006.docx]

**Point-by-Point Responses**

Dear editor,

We acknowledge the detailed evaluation of our study.

Bellow, you will find a point-by-point response answering reviewer’s comments. All changes are highlighted (in yellow) in the manuscript.

Sincerely,

Maria Laura Costa, MD, PhD

**Reviewer 1-**

One of the 2 referees who revised the paper suggested “REJECTION”, based on the fact that “the paper is primarily descriptive without any comparison to another approach to maternity care during the pandemic. Without an historical or actual comparison to assess whether the changes/programs instituted in the participating centers had any effect on clinical outcomes, we cannot determine whether such changes provided any benefit that could be used now or in the future.  In addition, the paper still requires revision into proper scientific English”and further stated “I am still unclear as to the intent of study/paper.”

**RESPONSE:**

We agree that the paper is mostly descriptive and that there is no comparison of time periods- this happened because it was submitted initially in September/2020, with data up to July/2021, during the first wave of the pandemic in Brazil. The aim of the study was to report the challenges in obstetrics and also in under resourced settings, with a quantitative and qualitative approach. The changes described are fundamental to ensure proper healthcare during the pandemic and provide adequate and safe environment both for patients and health workers and represented the prompt response of institutions in a timeframe of much uncertainty, lack of PPE (personal protective equipment) in many settings, need for extra ICU beds and resizing of emergency rooms and medical wards.

At the time of submission, we already pointed towards awareness regarding maternal deaths due to COVID-19 in Brazil and unfortunately this concern, reported in this manuscript with 15 maternal deaths in a 5 months period was further evidenced. National data report a total of 5575 cases of severe COVID-19 disease among pregnant and postpartum women in 2020, with 386 maternal deaths (6.9%) (<https://observatorioobstetrico.shinyapps.io/covid_gesta_puerp_br/>).

**Reviewer 2-**

The second referee only suggested minor changes.

**RESPONSE:** Thank you. Such comments were not reported.

**Editor´s suggestions and comments:**

My opinion is that the paper can contribute by providing description of some changes underwent by the 16 hospitals of the Rebraco initiative in the first period of the pandemic, but it needs major changes to be suitable for publication.   I am here trying to help the paper authors by suggesting some changes that in my view could make the paper more suitable for publication.

**RESPONSE:** Thank you. We acknowledge all suggestions and have worked to address them and improve the original submission.

1. The paper currently lacks a good structure, and element of internal consistency, therefore and difficult to follow. I would suggest to check well consistency in between the study objective sentence> methods > results > discussion. These must be logically aligned. For example, the study objectives in the abstract (lines 4-5) does not mention the qualitative component.

**RESPONSE:**

Thank you. We have made significant changes to address consistency throughout the manuscript. The adjusted sentences are highlighted. The aim of the study in the abstract was corrected:

“This study aims to describe the timeframe, implemented strategies and perspectives of health managers on the challenges to face the pandemic in 16 different maternity hospitals that comprise a multicenter study in Brazil, called REBRACO (Brazilian network of COVID-19 during pregnancy).”

2. I suggest adding the study design in the title, this will facilitate reading

**RESPONSE:**

The title was changed according to the suggestion, to include the study design

“Facing the COVID-19 Pandemic inside maternities in Brazil: a mixed-method study within the REBRACO initiative”

3. Suggestions to improve the abstract_  here, beside revising the sentence on the study objectives,  you need to add key elements currently lacking, such as: 1) study time period ; 2) sample size; 3) more quantitative data (eg data available in Table 2 and 3); 3) avoid in the abstract vague wording such as “Changes in infrastructure and staff were reported” (line 18) or “Training session were less common” (line 20), these sentence adds very little since they are very unspecific and lack quantitative data; 4) be consistent with your study methods, so report key findings both quantitative and qualitative component; 5) the conclusion sentence need to the refer to the study findings, please see other papers as example; a typical sentence would be “study findings suggest that hospital of the REBRACO initiatives underwent major changes…; fear etc (qualitative component). Please note that the abstract is one of the key section of the paper, therefore if you wish to resubmit please consider revising it carefully.

**RESPONSE:**

Thank you. All information was included/adjusted accordingly and detailed bellow in italic.

1) **study time period:** *from January to July/2020 (line 11);*
2) **sample size**: *for the qualitative study: “A qualitative study by purposeful and saturation sampling was undertaken with healthcare managers”; for the quantitative study, we considered all cases of COVID-19 reported by each participating center and their information on total number of deliveries, livebirth, maternal deaths.*
3) **more quantitative data (eg data available in Table 2 and 3. avoid in the abstract vague wording such as “Changes in infrastructure and staff were reported” (line 18) or “Training session were less common” (line 20), these sentence adds very little since they are very unspecific and lack quantitative data;**

*The required information was added:* *All maternities performed relocation of beds designated to labor ward, most (75%) acquired mechanical ventilators, only the minority (25%) installed new negative air pressure rooms. Considering human resources, around 40% hired extra health professionals and increased weekly workload and the majority (68.7%) also suspended annual leaves. Only one center implemented universal screening for childbirth. Qualitative results showed that main challenges experienced were related to the fear of the virus, concerns about reliability of evidence, lack of resources and clear need for mental health support among health professionals.*

4) **be consistent with your study methods, so report key findings both quantitative and qualitative component***- Improved as reported above.*
5) **the conclusion sentence need to the refer to the study findings, please see other papers as example; a typical sentence would be “study findings suggest that hospital of the REBRACO initiatives underwent major changes…; fear etc (qualitative component). Please note that the abstract is one of the key section of the paper, therefore if you wish to resubmit please consider revising it carefully**.

*Thank you. Conclusion now refers to main study findings:*

*“Study findings suggest that maternities of the REBRACO initiative underwent major changes in facing the pandemic, mostly resizing emergency rooms, relocating beds and adding extra ICU units and new ventilators. On human resources, most centers had to employ increased weekly workloads and listening to health managers, main concerns included fear of the virus and lack of resources. In the first months of the pandemic, there were 15 maternal deaths due to COVID-19 in the reported centers.”*

4. In order to improve the structure of the method section, I would suggest to add in the method section a paragraph for the quantitative component and one for the qualitative component. Both paragraphs would benefit of subparagraphs were all the following key element are clearly described: i) Population and setting, ii) data collection tools and methods, iii) data analysis.

**RESPONSE:**

Thank you. We have now organized methods in two subgroups (quantitative component and qualitative component) with the required information.

5. Please check carefully what belongs to each paragraph. For example, the current sentence and the beginning of the results section (lines 172-177) seems to be to pertain to the “study population” in the method section. It’s not clear if the sentence on the sample in lines 127-135 pertains to the quantitative or to the qualitative component.

**RESPONSE:**

Thank you. We agree, the first sentence in results belongs to methods and was excluded.

The information on informed consent for each component of the study is now more clear in the methods, with the previous suggestion of better defining quantitative and qualitative approaches (with sub-headings added).

6. The paper currently is very descriptive, often vague and/or not concise. A scientific paper needs to be concise and specific. Please if wishing to resubmit  revise text in order to be concise and specific. I suggest avoid in the paper text and in the abstract vague wording such as “data on several topics such as .. were collected” (line 102) and rather, make explicit exactly what is the list of variables which were collected (this could be done in a Supp Table). Grouping them consistently in methods and results (eg Infrastructures vs Human resources, as already done in Table 2) may also be helpful. Counting them is also helpful; for example, you may say in the method section that you collect data on XX key variable, covering the following domains: infrastructures (XX variables); human resources (XX variables); etc. This would be more concise and more specific that the current text.

**RESPONSE:**

Thank you. We have included all suggestions in the text and added the Supporting Box bellow with the data requested”. The paragraph now is:

“For the quantitative component, we collected data on the infrastructure/equipment of the units, maternal and perinatal health indicators, characteristics of service provision and modifications on staff and human resources. Detailed material can be found in the Supporting Information (S1)”.

**Supporting information-S1**

**S1: Variables requested for the characterization of the included REBRACO centers.** List of variables which were collected through online forms

**Infrastructure/Equipment**

Number of beds designated for labor ward (per month, from March to August)

Number of beds designated for obstetric medical ward (per month, from March to August)

Number of beds designated for intensive care (per month, from March to August)

Availability of the tests to confirm the infection by SARS-CoV 2 and number of tested women (per month, from March to August)

Implementation of modifications in the maternity´s infra-structure (resizing wards, relocation of beds, acquiring new equipments, such as ventilators)

Availability of PPE (Personal Protective Equipment)

**Maternal and perinatal health indicators**

Total number of attendances at the obstetric emergency care unit (per month, from March to August)

Number of attendances at the obstetric emergency care unit (per month, from March to August) for pregnant and postpartum women with suspected COVID-19

Number of new confirmed cases of COVID-19 in pregnant and postpartum women (per month, from March to August)

Criteria for suspected cases of COVID-19

Number of hospitalizations of pregnant and postpartum women in the unit (per month, from March to August)

Number of hospitalizations due to COVID-19 in pregnant and postpartum women in the unit (per month, from March to August) (suspected or confirmed)

Number of new cases of severe acute respiratory syndrome (SARS) in pregnant/postpartum women, in the unit (per month, from March to August)

Number of new cases of severe acute respiratory syndrome (SARS) caused by COVID-19 in pregnant/postpartum women, in the unit (per month, from March to August)

Number of maternal deaths due to all causes in the unit (per month, from March to August)

Number of maternal deaths due to COVID-19 unit (per month, from March to August)

Number of live births in you unit (per month, from March to August)

Cesarean section rate (%; elective or intrapartum cesarean) in the unit (per month, from March to August)

Number of live births in the municipality unit (per month, from March to August)

Number of stillbirths in the unit (per month, from March to August)

Number of stillbirths in the municipality unit (per month, from March to August)

**Other characteristics of service provision**

Referral center for cases suspected/confirmed for COVID-19 (y/n)

Rules on the presence of companions during childbirth (always, often, sometimes, rarely or never; per month, from March to August)

How long on average was the turnover to obtain test results for COVID-19? (in days; per month, from March to August)

Gynecological and obstetrical services according to the usual standard in the unit (suspended, reduction, no change, not applicable)

**Staff/Human Resources**

Presence of Ob&Gyn medical residents (y/n)

Number of professionals that received sick leave due to COVID-19 in the unit (per month, from March to August)

Members of the COVID-19 Emergency Action Committee (EAC) - (according to type of health professionals) and date of EAC implementation (from January to August)

Modification of work schedule of health professionals (Hiring new professionals, increase in weekly workload, suspension of annual leaves)

Content of the training program for COVID-19

7. Consistency between the method section and the results section in terms of variable reported need to be ensured. Also, consistency in the discussion on key findings of both components need to be there.

**RESPONSE:**

Thank you. We have revised the manuscript for consistency, included data that were not reported in tables (table 1 was revised) and better reported results and organized the discussion.

This paragraph is now referred to table 1, for example:

“All maternities established a local Covid-19 Emergency Action Committee (EAC) and implemented a local protocol for contingency and management of COVID-19 in pregnancy. The EAC was usually comprised of a general practitioner or internal medicine (73% of the centers, n=11), Obstetrics and Gynecology specialist (75%, n=12), intensive care specialist (68.7%, n=11), nurse (75%, n=12), member of the Infection Prevention and Control Committee (75%, n=12), administrative assistant (68.7%, n=11), and, less often, of a physiotherapist (31.2%, n=5) (Table 1). All the 16 maternities had access to diagnostic laboratory testing. However, only 6 maternities (37.5%) had resources to test any suspected case of COVID-19.”

8. Be careful in wording. For example, revise sentences such as “the observational mixed-method component” (line 102). Your study design is mixed methods, and in it you have a quantitative and a qualitative component.

**RESPONSE:**

Thank you. We have corrected the wording throughout the manuscript.

9. Avoid in the introduction section sentences which rapidly become outdated, such as data on number of people affected by COVID (lines 33-36 and 36-38). Either delete them of make explicit that they refer to your study period.

**RESPONSE:**

We agree and have modified the paragraph to avoid too many numbers and include only data consistent with the considered study period, in Brazil.

“Coronavirus disease 2019 (COVID-19) has threatened the world, since March 2020, when the pandemic was officially declared by the WHO (World Health Organization) [1]. Some countries were especially affected and faced individual challenges towards viral dissemination. Brazil was certainly one of those, recognized as a pandemic hotspot with more than 4.1 million people infected and over 126,000 deaths, in the first 6 months of the pandemic”

10. The discussion section is quite long. I would suggest to cut it down, delete redundancies and flag out key concepts. Typically, a discussion sections include the following paragraphs: 1) key findings/ what this study adds (very concise, not a repetition of results section), 2) interpretation when comparing to existing literature; 3) study limitations; 4) Recommendations for research 5) recommendations for policy makers

**RESPONSE:**

Thank you. We have reviewed discussion, excluded redundancies (three paragraphs) and better organized ideas, according to the suggestions above.

Now, the first paragraph addresses the key findings:

“The 16 maternities of the REBRACO initiative were mostly university hospitals, which underwent major changes and adaptations in facing the first few months of the pandemic, resizing emergency rooms, relocating beds and adding extra ICU units, with acquisition of ventilators. On human resources, most centers had to employ increased weekly workloads, deal with medical leave due to COVID infections, and hire extra health professionals. Listening to health managers, main concerns included fear of the virus and lack of resources. There was a clear limitation in the capacity of testing during the considered study period, less than half of the centers had resources to test any suspected case of COVID-19.”

We again, acknowledge all suggestions and hope that the revised version of the manuscript is suitable for publication.

Best regards,

Maria Laura Costa
