# Supplementary material for: Facing the COVID-19 pandemic inside maternities in Brazil: A mixed-method study within the REBRACO initiative
Source: PLoS One. 2021 Jul 23;16(7):e0254977. doi: 10.1371/journal.pone.0254977 (PMC8301675; doi:10.1371/journal.pone.0254977)
Supplement: Attachment — Submitted filename: Response_PloseOne_R3.docx [file pone.0254977.s007.docx]

**Point-by-Point Responses (PONE-D-20-29941R2)**

Dear editor,

We again acknowledge the detailed evaluation of our study. We agree that all previous suggestions and comments have greatly improved the manuscript: “Facing the COVID-19 Pandemic inside maternities in Brazil: a mixed-method study within the REBRACO initiative”.

Bellow, you will find a point-by-point response answering reviewer’s comments. All changes are highlighted (in yellow) in the manuscript.

Sincerely,

Maria Laura Costa, MD, PhD

**Reviewer 1-**

The paper has improved a lot, but need further improvement. Again, I will give here some suggestions on how to improve it:

ABSTRACT

Line 4: is the “the timeframe” really one of the indicators? Where is it in results? Otherwise delete from abstract and also from the text

**RESPONSE:** We agree, the term was confusing. We have deleted. The aim now is:

“This study aims to describe the strategies implemented and the perspectives of health managers on the challenges to face the pandemic in 16 different maternity hospitals that comprise a multicenter study in Brazil, called REBRACO (Brazilian network of COVID-19 during pregnancy).

It was also deleted from the text.

Line 7-10. Find a way to be more concise on quantitative indicators.

**RESPONSE:** Thank you. We have made it more concise. It now reads:

“Mixed-method study, with quantitative and qualitative approaches. Quantitative data on the infrastructure of the units, maternal and perinatal health indicators, modifications on staff and human resources, from January to July/2020. Also, information on total number of cases, and availability for COVID-19 testing. A qualitative study by purposeful and saturation sampling was undertaken with healthcare managers, to understand perspectives on local challenges in facing the pandemic.”

Line 12 versus line 4: either use the word “perspective” (line 4) or “local challenges” (line 12). The 2 words do not have the same meaning. Or should it be perspectives on local challenges?

**RESPONSE:** Thank you. We believe the most accurate would be “perspectives on local challenges”. We have changed the text.

Line 14: delete “Rebraco centers respond for a total of 4000 deliveries/months “ or move it to introduction

**RESPONSE:** It was deleted.

Line 21 “while most only tested” : once again I advise to avoid using unspecific words such as most

**RESPONSE:** Thank you. We have included the complete information:

“while around 60% of the centers only tested moderate/severe cases with hospital admission”

Conclusion: line 26-30 are just a repetition of study findings, delete these and add in conclusion recommendations for policy makers and for further research.

**RESPONSE:** Thank you. We have adjusted the conclusion in order to address main findings and recommendations for policy makers and for further research.

“Study findings suggest that maternities of the REBRACO initiative underwent major changes in facing the pandemic, with limitations on testing, difficulties in infrastructure and human resources. Leadership, continuous training, implementation of evidence-based protocols and collaborative initiatives are key to transpose the fear of the virus and ascertain adequate healthcare inside maternities, especially in low and middle-income settings. Policy makers need to address the specificities in considering reproductive health and childbirth during the COVID-19 pandemic and prioritize research and timely testing availability.”

INTRO

Line 42-45   are not relevant to your research question. Please revise to make clear what are the knowledge gaps related to HS and why is your research question relevant

**RESPONSE:** Thank you, it really was not clear. The sentence needs to present the challenge that the country size, economic and political crises can add to a pandemic. We have changed it to:

“Brazil is an upper-middle-income country of continental size, with great social and economic disparities among different regions, and major political crisis. These characteristics enhance the challenges in facing a pandemic”.

Line 59: you state that the REBRACO collect qualitative data from women experiences, but there is no further mention on this in the paper

**RESPONSE:** Thank you. We have adjusted the text to make sure that it is clear that we are describing the main study, and how the initiative was built, with the reference for the overall protocol (actually a Letter that describes the main objectives of the project, as a “call to action”). Therefore, in the current analysis we only included the qualitative data on health professionals.

“Overall, the REBRACO initiative aimed at evaluating the clinical, epidemiological and laboratory aspects related to SARS-CoV-2 infection during pregnancy, besides a qualitative assessment of women and professionals experiencing such a situation, to identify maternal and perinatal outcomes and collect relevant information to provide quick responses and proper organization of health services to confront the COVID-19 pandemic [11].

As part of the main study, we aim to describe the strategies implemented and the perspectives of health managers on the challenges to face the pandemic in 16 different maternity hospitals …”

Line 63-65. Add that you also collected perspectives of HW

**RESPONSE:** we have added the information on HW perspectives.

METHODS

Line 67-69 and 73-75 are a repetition of lines 54-57. I suggest to place the background information on REBRACO in the Introduction. Here in methods add details on study population and settings- Move here lines 165-169

**RESPONSE:** Thank you. We have deleted repeated information.

Line 76-78 and 99-101 repeated the study objectives again

**RESPONSE:** Repetition was deleted.

Line 102-132 and 133-162 These sections still lack some structure. Please first describe what data you collected, then how, and then how the analysis performed. Separate paragraphs so that they are clearly identified

**RESPONSE:** Thank you. We agree that information was not well organized. We have structured as suggested.

“For the quantitative component, we collected data on the infrastructure/equipment of the units, maternal and perinatal health indicators, characteristics of service provision and modifications on staff and human resources. All information was retrieved through an electronic form developed by the coordinating center and sent by e-mail to the Principal Investigator of each center (Health Manager, responsible for the Obstetrical Emergency Action Committee - EAC - in each institution). The data collection form is provided as Supporting Information (S1) and includes data on: characteristics of the maternities, their response program, their training program and some indicators related to the COVID-19 pandemic such as the number of cases, number of maternal deaths, maternal mortality ratio due to COVID-19 in the period, the time interval between the implementation of the response program and the first suspected COVID-19 cases in each center and the characteristics of the tests available in the obstetric units.

All suspected cases of COVID-19 in the considered maternities were assessed for informed consent and their data was only included after such consent, with identity kept confidential. Considering biosafety reasons, the study has authorization, by the Institutional Review Board, for both- written informed consent and consent by phone to use information from medical records and sample collection. Mostly suspected cases with hospital admissions have written informed consent, cases that were evaluated and tested in the emergency room, with no needed admission were later consented by phone. The researcher read and explained the consent form and assured that women could withdraw from the study without any interference with planned medical care. All interviews were recorded on audio after consent from the participants, using the Microsoft Skype platform.“

Table 1. there are only few quantitative indicators on your research question in this table, consider adding in tables data that now are in the text

**RESPONSE:** We have adjusted the Table description in order to present clearly the results:

“Table 1. Characteristics of included maternities in the REBRACO study (n=16), participating members of the local Emergency Action Committees (EAC) and information on COVID-19 infection and some healthcare indicators.”

CONCLUSION

Still very long, please reduce length

**RESPONSE:** it was reduced.

“Study findings suggest that maternities of the REBRACO initiative underwent major changes in facing the pandemic, with limitations on testing, difficulties in infrastructure and human resources. Leadership, continuous training, implementation of evidence-based protocols and collaborative initiatives are key to transpose the fear of the virus and ascertain adequate healthcare inside maternities, especially in low and middle-income settings. Policy makers need to address the specificities in considering reproductive health and childbirth during the COVID-19 pandemic and prioritize research and timely testing availability.”

We again, acknowledge all suggestions and hope that the revised version of the manuscript is suitable for publication.

Best regards,

Maria Laura Costa
